# 

**Published:** 1889-07

**Authors:** 


### International Dental Congress—Held in Paris.

An International Dental Congress will be held in Paris about September 1st prox. Quite extensive preparations have been made for this meeting and doubtless it will be an interesting occasion, one to be enjoyed by all who may be so fortunate as to be there. There are many Americans practicing dentistry in Europe, and for those of this side to meet them will be a mutual pleasure, and in addition to this it will be a great gratification to make the acquaintance of those of our profession of other nationalities.

In addition to professional matters there will be an aggregation of the world's wonders in the Paris Exposition, to see and examine which would more than repay the time and expense of a journey from any part of the world.

Paris is a world of itself, but when the rest of the world is added to it, certainly it is worth an effort to see it. In regard to the details for the journey, etc., we can not do better than to give the following from the *Dental Review*, Chicago, of June 15th.

"As a good many American dentists are likely to go to Paris this year to attend the Exposition incidentally, and the International Dental Congress particularly, a few suggestions may not be out of place, as to the time necessary to spend in going and coming, the cost of the journey, and such other hints as may prove useful to the tourist who crosses the ocean for the first time.

The Congress will open September first. Steamers leave Baltimore, Philadelphia, New Orleans, Montreal and Quebec,

Boston, and New York. Most of those who go will sail either from Montreal, Boston, or New York. The American Dental Association will meet at Saratoga the first Tuesday in August. This meeting will close in the afternoon of August 9th, giving ample time for any members to sail on Saturday the 10th, from New York, Boston, Philadelphia, or Montreal.

Steamers of the Anchor, National, Cunard, Transatlantic, and North German Lloyd lines will sail from New York on that day. A steamer of the Cunard line will sail from Boston, and one of the Allen line steamers will sail from Montreal. The following Tuesday a Guion line steamer may be taken, and on Wednesday and Thursday, White Star, Red Star, Hamburg, North German Lloyd, State, and Amsterdam and Rotterdam line steamers will depart. To one who goes on Saturday, August 10, more inducements will be offered than earlier or later. Special rates may be secured for that day from New York. The fare will range from $50 to $120, going, or return tickets may be had $90 to $200, all first-class, the only difference being in location of state rooms and the number of persons occupying them. Inside rooms are always a trifle cheaper than similar outside rooms. When two persons occupy a three-berth room they will have to pay extra for it. Our advice is, to one who has never been over, to take a steamer for Liverpool and return from Antwerp, Rotterdam, Amsterdam, Bremen, or Hamburg. The cost of railway travel will be saved one way from Liverpool to either of the places named. The journey may be reversed by sailing for a Belgian, Dutch, or German port, and coming home by the French line from Havre, or taking an English steamer from Glasgow, Liverpool, or London.

COST OF THE TRIP.

| | |
|---|---|
| Steamer from New York to Bremen or Hamburg, for one adult | $ 80. |
| Expenses on steamer, fees, etc | 10. |
| Expenses on land for 30 days, including second-class railway tickets from Hamburg via Berlin, Dresden, Cologne, Brussels, Paris, London, and Southampton or Liverpool | 180. |

| | |
|---|---|
| Ticket to New York.................................................. | 80. |
| Expenses on steamer, fees, etc....................................... | 10. |
| Total.................................................... | $360. |

Time going and coming about 18 to 20 days.

This will cover all the necessary expenses from New York to New York. The writer can do it comfortably for $300.

By taking a ticket on an Anchor or State line steamer $60 can be saved on the cost of the tickets. In going by the Red Star line or the Amsterdam line and returning by the Inman or White Star lines from Liverpool, a saving of $30 will be effected.

This is written for those who wish to economize and not for one to whom money is no object.

By writing to Thos. Cook & Son, 261 Broadway, New York, or E. M. Jenkins, New York, they will furnish tickets before you leave New York, by water and rail for the whole journey, and tickets for your meals in hotels wherever you stop. The hotel coupons will cost $2, $3, or $4 per day just as you choose. These coupons call for a room and two meals per day.

If you do not speak French or German it will cost about $1 per day more than we here estimate, on the Continent. We advise the taking of at least $100 more than you expect to spend, to guard against accidents or illness.

Buy a letter of credit, so you can draw money when and where you please. Purchase $20 or $30 of English, Dutch, French, or German money to carry in your pocket, so you will have something to spend when you land. Carry a cake of soap with you on the Continent, at least. Take plenty of old worn clothing to lie on deck with, including a traveling rug or blanket. Buy Cassell's Pocket Guide to Europe, or the Satchel Guide for 1889. Study the maps and plans and decide what you wish to see before you start. Badeker's Paris and his guide to London will more than pay for the small cost (about $2) if they are well studied. Articles not intended for personal use will have to be paid for when you arrive in New York at the usual import duty. You can attend the Congress without spending thirty days on land, but it will pay you to see something of the world while you are across the ocean and we strongly advise you to spend at least a week in London and a week in Paris, in addition to the week of the Congress. You could go as late as the 21st or 22d of August and arrive in time for the opening and return two days after the close of the Congress, the whole time from New York back to New York only occupying twenty-two or twenty-three days. We advise a longer stay."

### EDITORIAL.

### American Dental Association.

The twenty-ninth annual session of the American Dental Association will be held at Saratoga, August 6 to 9, inclusive. The promise is that this will be a very large and interesting and profitable meeting. This is the representative body of the profession of this country; it is its province to deal with all the more important interests of the profession; and there the real growth and progress that have been made appears, and may be made a common inheritance by all the other societies in the land, and especially those auxillary to the Association.

As usual, without doubt, many good and profitable papers will be read and discussed, and new things of all sorts be exhibited.

Let every one come prepared to contribute and to receive. This association has a work to do that can not be done elsewhere, and that fact ought to be fully recognized by all the members, and by all members of the profession who are interested in its welfare.

### The National Association of Dental Examiners.

ORANGE, N. J., JUNE 15TH, 1889.

The next meeting of the National Association of Dental Examiners will be held in Saratoga, N. Y., Tuesday, August 5th, 9:30 A. M., and at other times during the week, between the sessions of the American Dental Association. It is important to have every State Board represented.

FRED. A. LEVY, D.D.S., Secretary.

### The National Association of Dental Faculties.

INDIANAPOLIS, JULY 10, 1889.

The National Association of Dental Faculties will meet in annual session at Saratoga, New York, on Monday, August 5th, at 10 o'clock A. M., the exact place of assembly to be announced later. In August, 1888, this association ordered that thereafter delegates should be required to present duly executed credentials. Delegates are urged to report early on the date specified. Deans or secretaries of the colleges interested will please at once forward to the Secretary of the Association of Faculties single copies of their respective announcements for 1889-90.

By direction of, O. A. HUNT, President.
JUNIUS E. CRAVENS, Secretary.

### American Dental Association.

The twenty-ninth annual meeting of the American Dental Association will be held at Saratoga Springs, commencing at 10 o'clock A. M. Tuesday, August 6, 1889.

GEO. H. CUSHING, Rec. Sec'y.

### THE DENTAL REGISTER,

**A MONTHLY MAGAZINE DEVOTED TO THE INTERESTS OF THE**

### DENTAL PROFESSION.

*Terms Reduced to $2.00 Per Year. Single Copies, 25 Cents.*

**EDITED BY PROF. J. TAFT, D.D.S.**

—AND—

**Published by SAM'L A. CROCKER & CO., Ohio Dental and Surgical Depot.**

The volume commences in January and closes in December.

The REGISTER being issued monthly is always fresh, therefore Indispensable to the practitioner who desires to keep posted up with the new inventions and improvements which are daily developed. Neither expense nor effort will be spared to give value and variety to its contents. Articles will be illustrated with well executed engravings whenever they will add to the interest or assist to explanation.

Its extensive circulation and frequency of issue make it one of the BEST DENTAL ADVERTISING MEDIUMS in the United States.

All subscriptions must begin with January or July.

Local or special notices, five lines or less, will be inserted for $3 each insertion; over five lines, 50 cts. per line.

All advertising bills payable in advance, or at furthest, after the issue of the first number containing the advertisement. All transient advertisements to be paid for in advance.

☞CONTRIBUTIONS SOLICITED.

Exclusively Editorial Communications should be addressed to the Editor.

The latest number of the REGISTER may be ordered with or without other goods at any time, and all letters should be addressed to

**SAM'L A. CROCKER & CO., Ohio Dental and Surgical Depot, Cincinnati, O.**

